# *WRAP53* is Downregulated in Acute Myeloid Leukemia Patients and Positively Correlates With *HTERT* Expression

**DOI:** 10.1002/cnr2.70464

**Published:** 2026-02-08

**Authors:** Renan Brito Gadelha, Beatriz Maria Dias Nogueira, Caio Bezerra Machado, Flávia Melo Cunha de Pinho Pessoa, Anna Karolyna da Costa Machado, Germison Silva Lopes, Paulo Henrique Silva Rodrigues, Henrique Girão Martins, Deivide de Sousa Oliveira, Rodrigo Monteiro Ribeiro, Ricardo Parente Garcia Vieira, Manoel Odorico de Moraes Filho, Maria Elisabete Amaral de Moraes, André Salim Khayat, Caroline Aquino Moreira‐Nunes

**Affiliations:** ^1^ Department of Medicine, Clinical Genetics Laboratory, Drug Research and Development Center (NPDM) Federal University of Ceará Fortaleza Brazil; ^2^ Department of Hematology, Fortaleza General Hospital Dr. César Cals (HGCC) Fortaleza Brazil; ^3^ Department of Hematology, Fortaleza General Hospital (HGF) Fortaleza Brazil; ^4^ Department of Hematology, Hospital and Maternity São Vicente de Paulo (HMSVP) Barbalha Brazil; ^5^ Department of Biological Sciences, Oncology Research Center Federal University of Pará Belém Brazil; ^6^ Clementino Fraga Group, Central Unity, Genomics and Molecular Biology Laboratory Fortaleza Brazil

**Keywords:** acute myeloid leukemia, biomarkers, DNA repair, gene expression, *WRAP53*

## Abstract

**Background:**

Acute myeloid leukemia (AML) is a prevalent hematologic malignancy in adults, marked by clonal disorders in hematopoietic cells, rapid progression, and genetic heterogeneity. The *WRAP53* gene, which is associated with genomic stability due to its involvement in activities, such as DNA repair, TP53 regulation, and association with telomerase (hTERT), was the focus of this study.

**Aims:**

This study aimed to identify new potential molecular markers with prognostic value, based on specific targets, in order to contribute to a more accurate stratification of patients.

**Methods and Results:**

We assessed *WRAP53* and *hTERT* expression in 110 AML patients classified according to World Health Organization (WHO) guidelines. Using real‐time quantitative PCR, we investigated their expression and correlation with clinical outcome variables. *WRAP53* expression was significantly decreased in AML patients compared to controls, whereas we did not detect differences in *hTERT* expression. Correlation analysis revealed a moderate positive relationship between *WRAP53* and *hTERT* expression. Concerning the clinical parameters analyzed, significant differences were observed for *WRAP53* in terms of sex and age, whereas for *hTERT*, no differences in the parameters analyzed were observed. Overall survival analysis did not reveal a significant difference for either *WRAP53* or *hTERT*. The results presented demonstrate a downregulation of *WRAP53* in the studied sample and that, furthermore, the expression of the *WRAP53* and *hTERT* genes was correlated. In addition, the expression of *hTERT*, which is already indicated as a biomarker in AML, could not be correlated with the clinical characteristics analyzed in this study.

**Conclusion:**

We also suggest that the low expression of *WRAP53* may be associated with other mechanisms in AML, such as DNA repair, thus becoming a possible new promising molecular biomarker related to genomic stability in AML.

## 1 Introduction

Cancers arise through a series of mutations or genetic alterations that give the cell the ability to override proapoptotic and antiproliferative signals, allowing it to reach hallmarks such as replicative immortality, invasion, and metastasis [1, 2, 3].

Acute myeloid leukemia (AML) is a heterogeneous group of hematopoietic neoplasms that affect myeloid precursor cells and is characterized by the clonal expansion of immature leukemic blasts, primarily in the bone marrow that may subsequently reach the peripheral blood. The disease is characterized by high molecular heterogeneity and is associated with a poor prognosis, with an aggressive clinical course, low remission rates, and high mortality rates [4, 5].

Owing to the high mutational burden present in leukemias resulting from telomere attrition, chromosomal translocations are among the most common genetic alterations reported in this disease and play crucial roles in progression and resistance to treatment. Telomerase (*hTERT*) is the enzyme responsible for preserving telomere length at the ends of chromosomes; mutations in genes related to telomeres and telomerase, such as dyskerin pseudouridine synthase 1 (*DKC1*), telomerase RNA component (*TERC*), telomerase reverse transcriptase (*TERT*), *WRAP53*, and NOP10 ribonucleoprotein (*NOP10*), among others, which play a role in telomerase function and telomere maintenance, may contribute not only to genomic instability but also to bone marrow failure, increasing the risk of developing AML [5, 6, 7, 8].

In AML, alterations in *hTERT* expression have been reported [9, 10]. However, low or absent *hTERT* expression has also been observed in some patients [11], which may reflect the presence of alternative telomere maintenance mechanisms, such as ALT (alternative lengthening of telomeres), or indicate distinct molecular profiles within the heterogeneous landscape of AML [12]. This variation in expression makes the study of *hTERT* relevant for both diagnostic and prognostic purposes, especially when associated with other genes involved in genomic stability.

The *WRAP53* gene, located on chromosome 17p13, has three distinct transcriptional start sites, which give rise to three main transcript variants: WRAP53‐α, WRAP53‐β, and WRAP53‐γ [13]. Alternative splicing is a crucial process in the regulation of gene expression and allows a single gene to encode multiple functionally distinct protein products with opposing roles in physiological and pathological contexts [14, 15, 16, 17, 18]. This molecular mechanism confers on the *WRAP53* gene the ability to produce isoforms with specific subcellular locations and functions.

The isoforms: WRAP53α, WRAP53β, and WRAP53γ have different functions. WRAP53α is in charge of encoding a p53 regulatory RNA, whereas WRAP53β is a scaffold protein involved in the repair of DNA damage, the maintenance of nuclear bodies known as Cajal bodies, and the assembly of telomerase. There are still no studies reporting WRAP53γ function. WRAP53β cellular localization together with *WRAP53* expression in solid tumors are factors that predict an effective response to radiotherapy, in addition to serving as a tumor biomarker [8, 19, 20, 21, 22, 23]. Although there are no articles in the literature elucidating the function of *WRAP53* in leukemias, changes in its expression levels and alterations at the genetic level, such as mutations, have already been well reported in solid tumor neoplasms.

Therefore, the aim of this study was to evaluate the gene expression profiles of *WRAP53* and *hTERT* in a cohort of AML patients, to evaluate their possible roles in the leukemogenesis pathway.

## 2 Materials and Methods

### 2.1 Biological Samples

A total of 110 adult patients diagnosed with acute myeloid leukemia treated at General Hospital of Fortaleza, Dr. César Cals General Hospital, and São Vicente de Paulo Maternity Hospital, reference centers for oncohematological treatment in the state of Ceará (Ceará, CE, Brazil) participated in this study. To categorize patients into different groups, the European LeukemiaNet (ELN) guidelines [24] were used, where patients were categorized according to age, white blood cell (WBC) count, hemoglobin levels, and sex. The cytogenetic risk assessment was carried out according to the World Health Organization (WHO) criteria [25]. Furthermore, previously, patients in this study were screened for genetic abnormalities, such as *BCR::ABL1* p190, *PML::RARA, RUNX1::RUX1T1*, and *CBF::MYH11*, via reverse transcription polymerase chain reaction (RT–qPCR) [26]. Additionally, 10 peripheral blood samples were collected from healthy volunteers and employed as control samples. Although all patients in the study were adults, their legal guardians informed them about the informed consent in cases where the patient's condition or status prevented personal signature, after which the patients underwent a clinical evaluation for the collection of biological material. The methods were performed in accordance with the Helsinki guidelines and regulations. The study was approved by the Ethics Committee of the Federal University of Ceará and Dr. César Cals General Hospital with the following approval numbers: 4.339.719 and 5.823.921.

### 2.2 RNA Extraction and cDNA Conversion

Total RNA was extracted from the peripheral blood buffy coats of AML patients via the TRIzol Reagent (InvitrogenTM) according to the manufacturer's instructions and quantified via absorbance using a NanoDrop spectrophotometer (Thermo Fisher Scientific).

Complementary DNA (cDNA) was synthesized from 20 ng of total RNA extracted via a high‐capacity cDNA reverse transcriptase kit (Thermo Fisher Scientific). The conversion step was carried out in a Veriti thermal cycler (Applied Biosystems). The cDNA samples were stored in a −20°C freezer to establish a cDNA library and remained frozen until analysis.

### 2.3 Validation of Gene Expression by Real‐Time Quantitative Polymerase Chain Reaction (qPCR)

The genes selected for evaluation of gene expression were *WRAP53* (Hs_Hs00216360_m1), *hTERT* (Hs_00972650_m1), and the endogenous *ABL1* gene (Hs01104728_m1), which was used as an internal control. The detection method was the TaqMan gene expression assay system (Applied Biosystems, Foster City, CA, USA), and qPCR was performed via the QuantStudio5 Real‐Time PCR system (Applied Biosystems, Foster City, CA, USA). The *ABL1* reference gene used in this study was previously validated by RT–qPCR detection in AML patients and was determined to be one of the most appropriate endogenous genes for expression assay analysis [27]. Among the different transcripts of the *WRAP53* gene, the one analyzed in this study was WRAP53β.

For each sample, the following were used: 1 μL of cDNA, 0.5 μL of each primer/probe, 5 μL of TaqMan Gene Expression Master Mix (Life Technologies, Carlsbad, CA, USA), and 3.5 μL of ultrapure water. Gene expression levels were based on absolute and relative analyses via the 2‐ΔΔCQ method, with samples from healthy donors used as calibrators/controls. Each sample was analyzed in replicate for experimental and technical validation, following international standards for evaluating gene expression via real‐time PCR [28]. Fold change (FC) criteria were defined to identify differentially expressed genes. For the *WRAP53* gene, two groups were created: one with FC values < −1 (differentially expressed) and another with FC values between −1 and 1 (not differentially expressed). For *hTERT*, the categorization included three groups of values: FC < −1, between −1 and 1, and greater than 1 (differentially expressed).

### 2.4 Statistical Analysis

The data on the relative mRNA expression of *WRAP53* and *hTERT* are expressed as the means to determine the potential associations between gene expression and variables. Normality was assessed by the Kolmogorov–Smirnov or Shapiro–Wilk test and was applied to each specific group. The outliers were removed on the basis of the upper and lower fences of the interquartile range. Differences in continuous variables were analyzed via the Mann–Whitney or unpaired *t*‐test to compare medians between two groups and the Kruskal–Wallis test or one‐way ANOVA to compare medians among variables with three or more groups. The correlation between two continuous variables was determined via the Pearson correlation coefficient. Furthermore, a multiple linear regression was performed to evaluate the association between *WRAP53* and *hTERT* expression, simultaneously adjusting for clinical variables. Survival probabilities were estimated via the Kaplan–Meier method, and differences in survival distributions were assessed via the log‐rank test, where overall patient survival was determined from the date of diagnosis until death or the last follow‐up. Statistical analyses, along with graph generation, were carried out via GraphPad Prism (version 8.0) software. The LogRank test was performed with Jamovi software (version 2.3.28). Significant differences were considered with a 95% confidence interval (*p* < 0.05).

## 3 Results

### 3.1 Clinical Features of AML Patients

In total, samples were collected from 110 patients, 56 (50.9%) men and 54 (49.1%) women. The average age among patients was 50.3 years, with men averaging 49.3 years and women 51.4 years. The clinical characteristics of AML patients are detailed in (Table 1). Key findings include low hemoglobin levels in 65.4% of patients, circulating blasts in 59%, and thrombocytopenia in 84.5%. Immunophenotyping revealed predominant markers such as CD33, CD117, and CD45, while karyotype analysis classified 44% of patients as adverse. Patients for whom karyotype and fusion results were unavailable and not detected in previous screening were classified as not classifiable (NC).

**TABLE 1:** Clinical features of patients with AML.

AML patients' clinical data	
Hemoglobin	< 8 g/dL: 72 (65.4%) 8–10 g/dL: 29 (26.3%) > 10 g/dL: 9 (8.3%)
WBC	≤ 10.000/mm^3^: 47 (42.7%) > 10.000/mm^3^: 46 (41%) > 100.000/mm^3^: 18 (16.3%)
Blasts in peripheral blood	Yes: 65 (59%) No: 45 (41%)
Blasts in bone marrow	Blasts ≤ 10%: *n* = 9 Blasts ≤ 70%: *n* = 39 Blasts > 70%: *n* = 59 N.R: 3
Platelets	< 150.000/mm^3^: 93 (84.5%) > 150.000/mm^3^: 17 (15.5%)
LHD	LDH ≤ 400 μL: 37 LDH > 400 μL: 32 LDH > 1.000 μL: 13 N.R: 28
Immunophenotyping	CD33, CD117, CD45, CD34, CD13, HLA‐DR, MPO e CD64
Karyotype	Favorable: 9% *n* = 10 Intermediary: 30% *n* = 34 Adverse: 44% *n* = 48 N.C: 17% *n* = 18

Abbreviations: LDH, lactate dehydrogenase; N.C, not classifiable; N.R, no results; WBC, white blood cell.

### 3.2 Gene Expression of *WRAP53* in AML Patients

The analyzed data were normalized, and Log2 FC levels were used as a cutoff for the analysis of *WRAP53* gene expression. Patients were divided into two main groups; the first group consisted of samples with FC ≤ −1 (*n* = 79), representing reduced *WRAP53* gene expression, and the second group included samples with FC values between −1 and > 1 (*n* = 17), representing minimal variation or no significant change in *WRAP53* expression. For analysis of the expression results, patients were classified into different groups based on the ELN guidelines [24], being, by age ≤ 60 and > 60, in the leukocyte count (WBC), separated into groups of < 10 × 10^3^/mm^3^, > 10 × 10^3^/mm^3^, and ≥ 100 × 10^3^/mm^3^, in sex (male or female), and by hemoglobin levels < 8 g/dL, 8–10 g/dL, and ≥ 10 g/dL. For the cytogenetic risk group, the classification of patients was based on the WHO guidelines [25].

**FIGURE 1:**
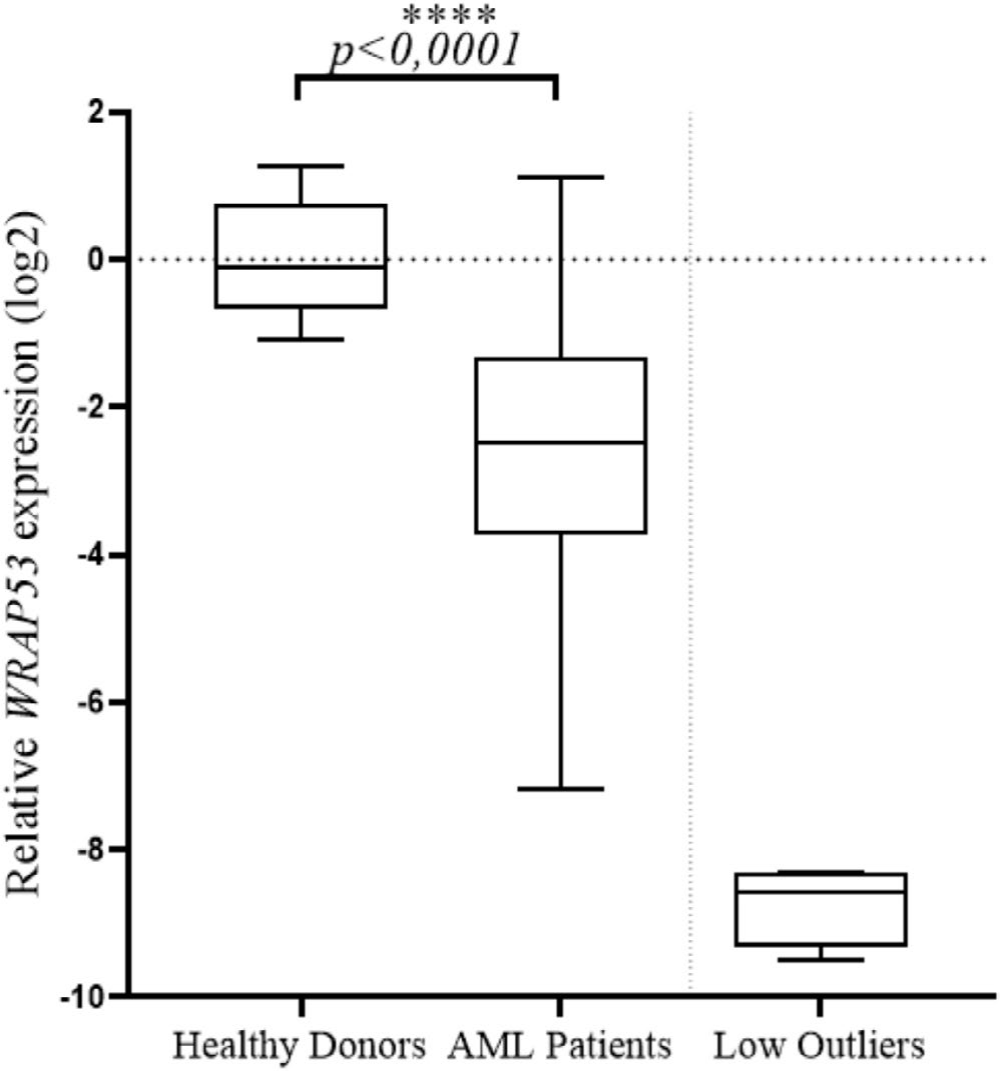
Evaluation of the expression of the *WRAP53* gene in peripheral blood samples from patients with AML compared with the control group. AML patients demonstrate a low expression of *WRAP53* compared to the control group (*n* = 10) (*p* < 0.0001), low outliers removed (*n* = 4). Data are presented as mean. For statistical analysis, normal distribution was assessed by the Kolmogorov–Smirnov normality test and was followed by the unpaired *t*‐test.

In our cohort of AML patients, the mean expression level of *WRAP53* was −2.490 and was lower than that in blood samples from 10 healthy donors (control group); the mean expression level in the control group was −0.1111 (*p* < 0.0001) and can be seen in Figure 1 where the entire studied sample is represented. WRAP53 was expressed in 100 of the 110 AML patients included in the study. In the 10 cases without *WRAP53* detection, expression of the endogenous *ABL* gene was confirmed. The outliers were not considered in the statistical calculations (*n* = 4).

When clinical variables such as sex, age, hemoglobin, leukocyte count, or cytogenetics were analyzed, statistically significant differences were observed across the sex and age groups. Furthermore, a slight trend was observed in some parameters, such as a greater percentage of men, a greater number of patients aged < 60, patients with hemoglobin below 8 g/dL, and a greater number of patients classified as having adverse risk in the FC < −1 groups.

In the male group (49 patients), the mean expression of *WRAP53* was significantly lower in cases with differential expression (FC ≤ −1) compared to the female group (47 patients), with values of −3.222 and −2.613, respectively (*p* = 0.0322). There was no significant difference in the group without differential expression (FC between −1 and 1). Regarding age, patients over 60 years old showed higher mean expression of *WRAP53* in the FC ≤ −1 group (*p* = 0.0496), but there was no significant difference in the FC between −1 and 1 group (*p* = 0.0795). (Table 2).

**TABLE 2:** *WRAP53* expression according to clinical features of AML.

Parameter	Total (*n* = 96)	*WRAP53* differentially expressed	*p*	*WRAP53* not differentially expressed	*p*
Sex					
Male (%)	49 (51)	−3222 (*n* = 43)	0.0322*	−0.0305 (*n* = 6)	0.6132
Female (%)	47 (49)	−2613 (*n* = 36)		−0.1975 (*n* = 11)	
Age					
≤ 60	68	−3120 (*n* = 57)	0.0469*	−0.3334 (*n* = 11)	0.0795
> 60	28	−2490 (*n* = 22)		0.218 7 (*n* = 6)	

*Note:* Distribution of clinical characteristics of AML patients with *WRAP53* expression, showing the comparison between differentially expressed (FC ≤ −1) and not differentially expressed groups (FC between −1 and 1). Data include sex, age, and mean values, along with their respective *p* values for statistical analysis, followed by the unpaired *t*‐test. Asterisks indicate significant differences.

For white blood cells (WBC), there was no statistically significant difference in *WRAP53* expression between the FC ≤ −1 and FC between −1 and 1 groups, regardless of WBC count (*p* = 0.3683 and 0.1953). Regarding hemoglobin levels, no significant association was observed between hemoglobin ranges and *WRAP53* expression (*p* = 0.1205 and 0.2637). In risk stratification, there was no significant difference in *WRAP53* expression among favorable, intermediate, adverse, and unclassified risk groups (*p* = 0.6198 and 0.4586).

### 3.3 Kaplan–Meier Survival Analysis for *WRAP53*

**FIGURE 2:**
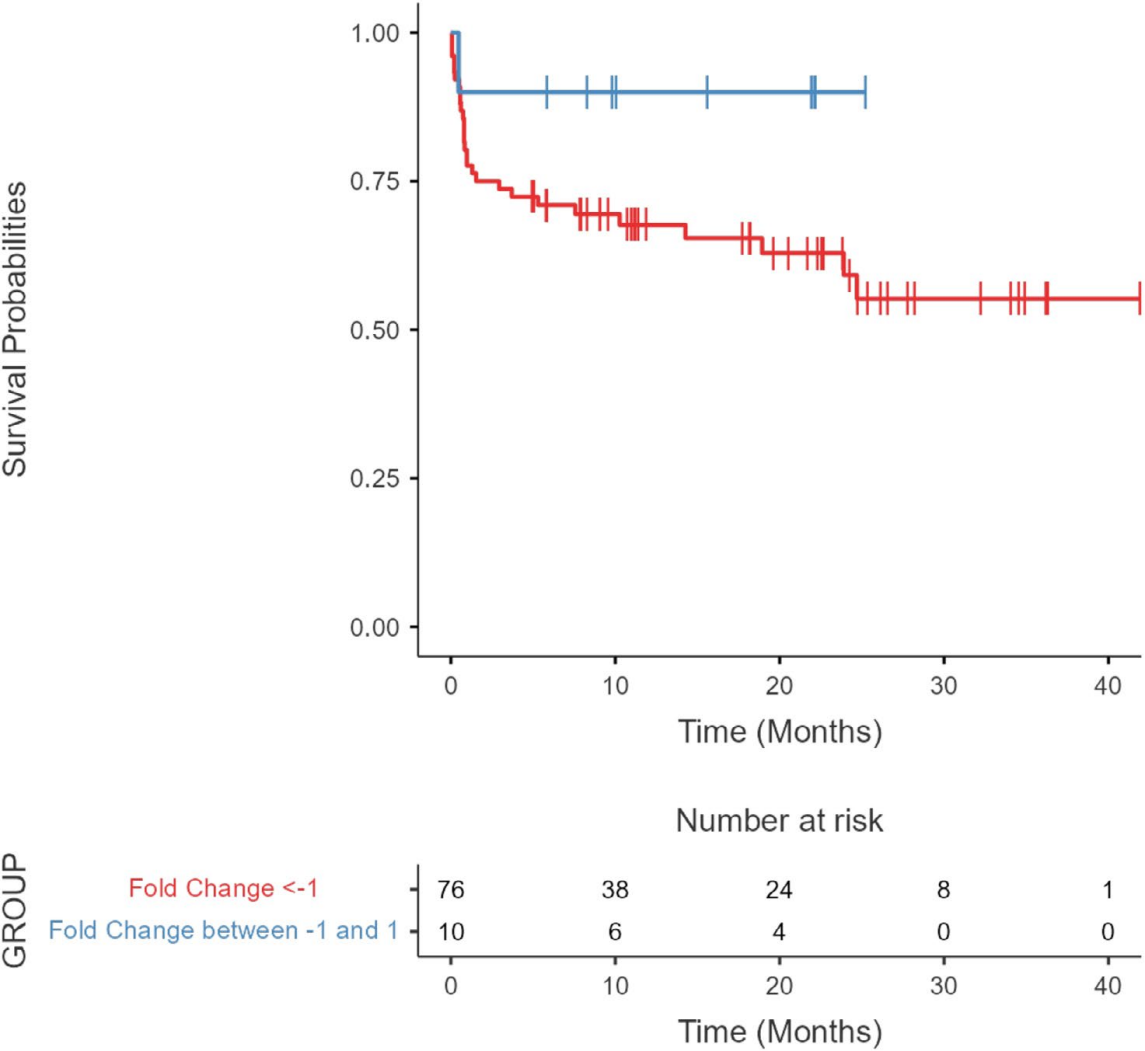
Survival analysis in patients with different levels of *WRAP53* expression. A Survival analysis of patients with different levels of *WRAP53* expression. There were no statistical differences between groups Log‐Rank (*p* = 0.150). The data shown in the risk table that is presented in the image shows the probability of the event (death) over time in the studied sample with information on the number of individuals at risk of experiencing the event at each time interval.

We performed a survival analysis to evaluate the association of *WRAP53* gene expression levels and their potential association with prognosis in hematologic malignancies. The analysis included all patients with complete clinical and epidemiologic data and who had *WRAP53* expression (*n* = 86). A The mean follow‐up time was 13.4 months. We used the same expression data, divided into two groups: patients with FC ≤ −1 and FC between −1 and 1 of *WRAP53* on the hypothesis that altered expression of this gene may influence the survival of patients with AML. The analysis showed that *WRAP53* expression levels did not present significant changes between the groups above or below the median Log‐Rank (*p* = 0.150), as shown in Figure 2.

### 3.4 Gene Expression of *hTERT* in AML Patients

As previously described, the data analyzed here were normalized, assuming that Log2 FC levels were used as a cutoff point, where patients were divided into three groups representing the variation in gene expression. The first group included samples with FC ≤ −1 (*n* = 38), indicating a reduction in *hTERT* gene expression; the second group included those with FCs between −1 and 1 (*n* = 26), representing minimal variation or no significant change in expression; and the third group consisted of those with FC > 1 (*n* = 13), indicating a variation in increased *hTERT* gene expression. For analysis of the expression results, patients were classified as in the previous analysis considering age, leukocyte count, sex, hemoglobin level, and cytogenetic risk group [24, 25].

**FIGURE 3:**
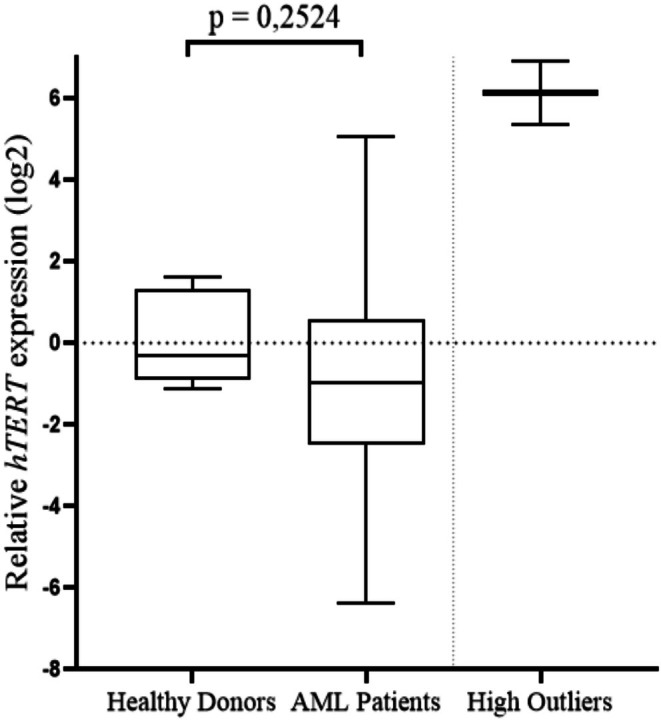
Evaluation of *hTERT* expression in the peripheral blood of patients with AML compared with the control group. In this study, we did not identify differences in *hTERT* expression (*p* = 0.2524) in our cohort compared with the control group, high outliers removed (*n* = 2). Data are presented as mean. For statistical analysis, normal distribution was assessed by the Kolmogorov–Smirnov normality test and was followed by the unpaired *t*‐test.

In this study, we did not identify differences in *hTERT* expression in our cohort of AML patients (mean *hTERT*: −0.9658) compared with blood samples from 10 healthy donors (control group, mean control group: −0.2942), *p* = 0.2524. Figure 3, which represents the entire sample studied, shows the expression level of the entire sample. *hTERT* expression was identified in 79 of the 110 patients with AML included in the study. In the 31 cases without *hTERT* detection, the expression of the endogenous *ABL* gene was confirmed. The outliers were not considered in the statistical calculations (*n* = 2).

The evaluation of clinical variables did not show statistical significance. Regarding risk stratification, patients classified as adverse risk were more likely to have a lower *hTERT* expression, although no significant difference was found for favorable or intermediate risk groups. Overall, although some trends were observed, these findings did not reveal statistical significance against clinical parameters.

The expression of the *hTERT* gene was compared between men and women with AML in the groups with FC ≤ −1, FC between −1 and 1, and FC > 1. In the male group (*n* = 38), the mean expression values were −2.591, 0.0342, and 3.232, respectively, while in the female group (*n* = 41), the mean values were −2.419, 0.4605, and 3.284, respectively. The *p* values were 0.8192, 0.6049, and 0.5338, indicating no statistically significant difference between sexes in the analyzed groups. Regarding age, there was also no significant difference in *hTERT* expression between individuals ≤ 60 years and > 60 years (*p* = 0.3382, 0.8410, and 0.8111 for FC ≤ −1, FC between −1 and 1, and FC > 1, respectively) (Table 3).

**TABLE 3:** *hTERT* expression according to clinical features of AML.

Parameter	Total (*n* = 77)	*hTERT* differentially expressed ≤ −1	*p*	*hTERT* not differentially expressed	*p*	*hTERT* differentially expressed > 1	*p*
Sex							
Male (%)	37 (49)	−2591 (*n* = 23)	0.8192	0.0342 (*n* = 8)	0.6049	3232 (*n* = 6) 3284 (*n* = 7)	0.5338
Female (%)	40 (51)	−2419 (*n* = 15)		0.4605 (*n* = 18)			
Age							
≤ 60	53	−2458 (*n* = 29)	0.3382	0.3651 (*n* = 17)	0.8410	3232 (*n* = 7)	0.8111
> 60	24	−2662 (*n* = 9)		0.3173 (*n* = 9)		3422 (*n* = 6)	

*Note:* Distribution of clinical characteristics of AML patients with *hTERT* expression, showing the comparison between low expression (fold change ≤ −1) and differentially expressed groups (fold change between −1 and 1) and moderate expression (fold change > 1). Data include sex and age with their respective *p* values for statistical analysis, followed by the Mann–Whitney test.

For the clinical variables analyzed regarding the *hTERT* gene, no significant differences in expression were observed between the groups with FC ≤ −1, FC between −1 and 1, and FC > 1. Regarding white blood cell (WBC) count, the *p*‐values were 0.1688, 0.7441, and 0.7308, respectively, indicating no statistical difference between the groups. For hemoglobin levels, there were also no significant differences in the ranges < 8, 8–10, and ≥ 10 g/dL (*p* = 0.3131, 0.1098, and 0.8678, respectively). In cytogenetic risk stratification, no significant differences were found between the groups (*p* = 0.0549, 0.4628, and 0.4628, respectively).

### 3.5 Kaplan–Meier Survival Analysis for *hTERT*

**FIGURE 4:**
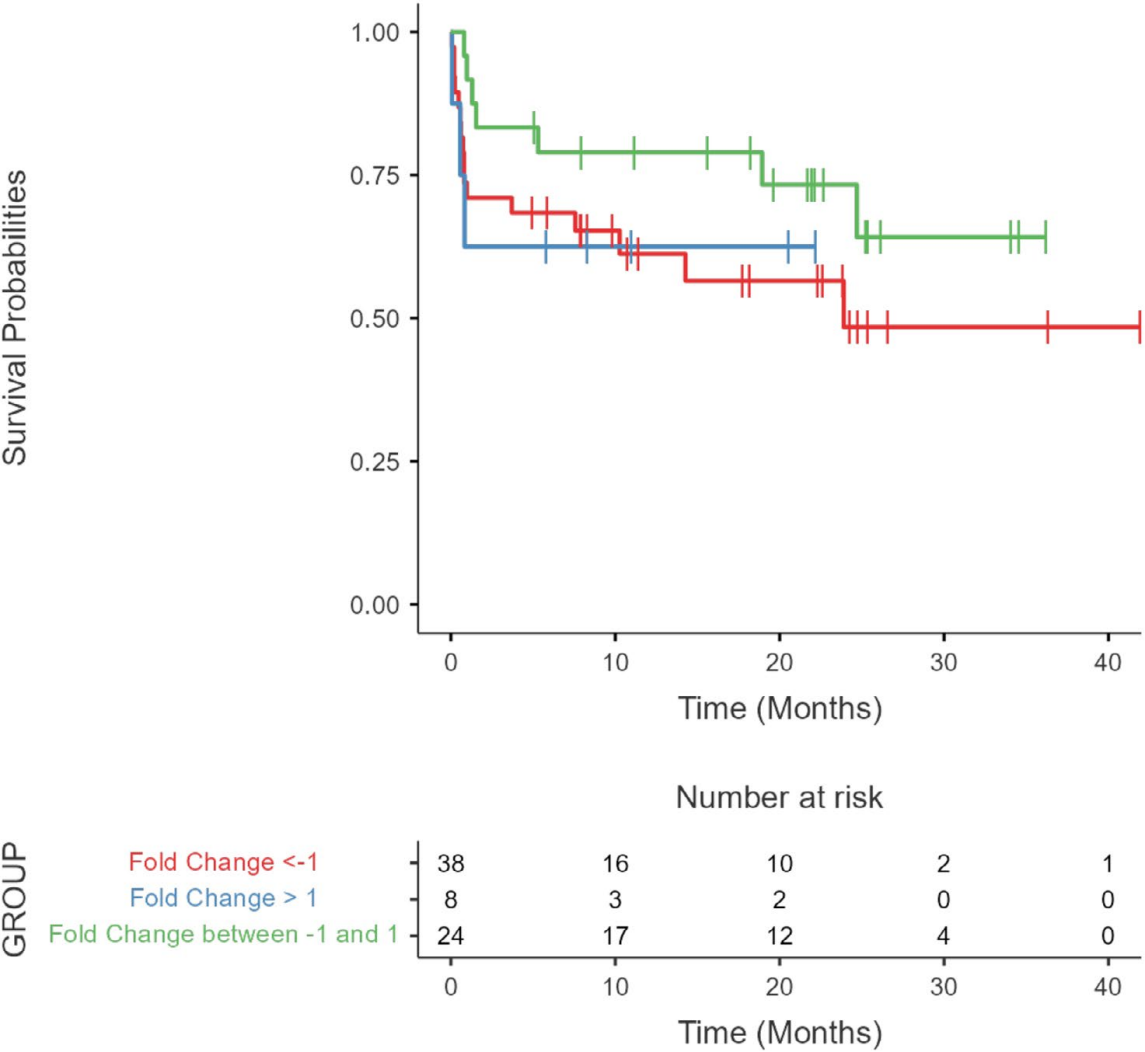
Comparison of survival time in patients with different levels of *hTERT* expression. Survival time was not statistically significant between expression groups (*p* = 0.316). The data in the risk table presented in the image show the probability of the event (death) over time in the studied sample with information on the number of individuals at risk of experiencing the event at each time interval.

We performed a survival analysis to assess the association of *hTERT* gene expression levels and their potential association with prognosis. The analysis included all patients with complete clinical and epidemiologic data and who had *hTERT* expression (*n* = 70). The median follow‐up time was 13.4 months. We used the same expression data, divided into three groups by FC values, namely: FC < −1, FC between −1 and 1, and FC > 1 on the hypothesis that altered expression of this gene may influence the survival of patients with AML. The analysis showed that *hTERT* expression levels did not present significant changes between the Log‐Rank groups (*p* = 0.316), as shown in Figure 4.

## 4 *WRAP53* Expression Is Correlated With *hTERT*

**FIGURE 5:**
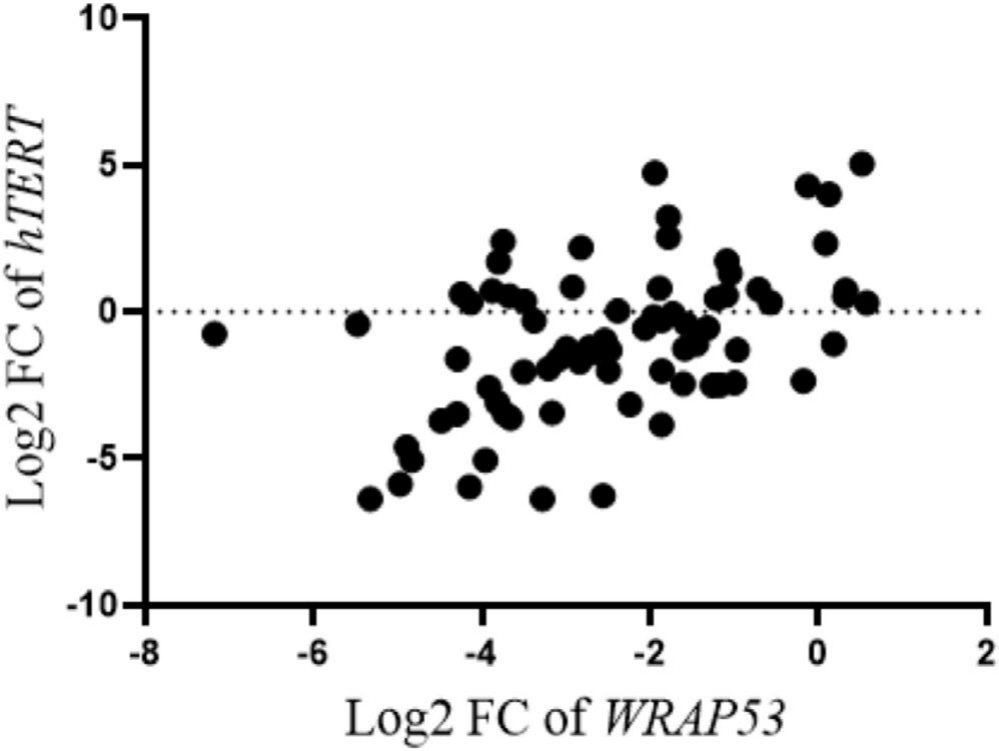
Correlation between *WRAP53* and *hTERT* gene expression in AML patients. The scatter plot shows the relationship between the log2 FC values of *WRAP53* and *hTERT*. Each point represents expression data. The analysis suggested a positive correlation between *WRAP53* and *hTERT* expression (*r* = 0.475, *p* < 0.0001).

A Pearson correlation analysis revealed a moderate positive relationship between *WRAP53* and *hTERT* expression levels, with a correlation coefficient of *r* = 0.475 and a *p* value < 0.0001, indicating a statistically significant positive association (Figure 5).

**FIGURE 6:**
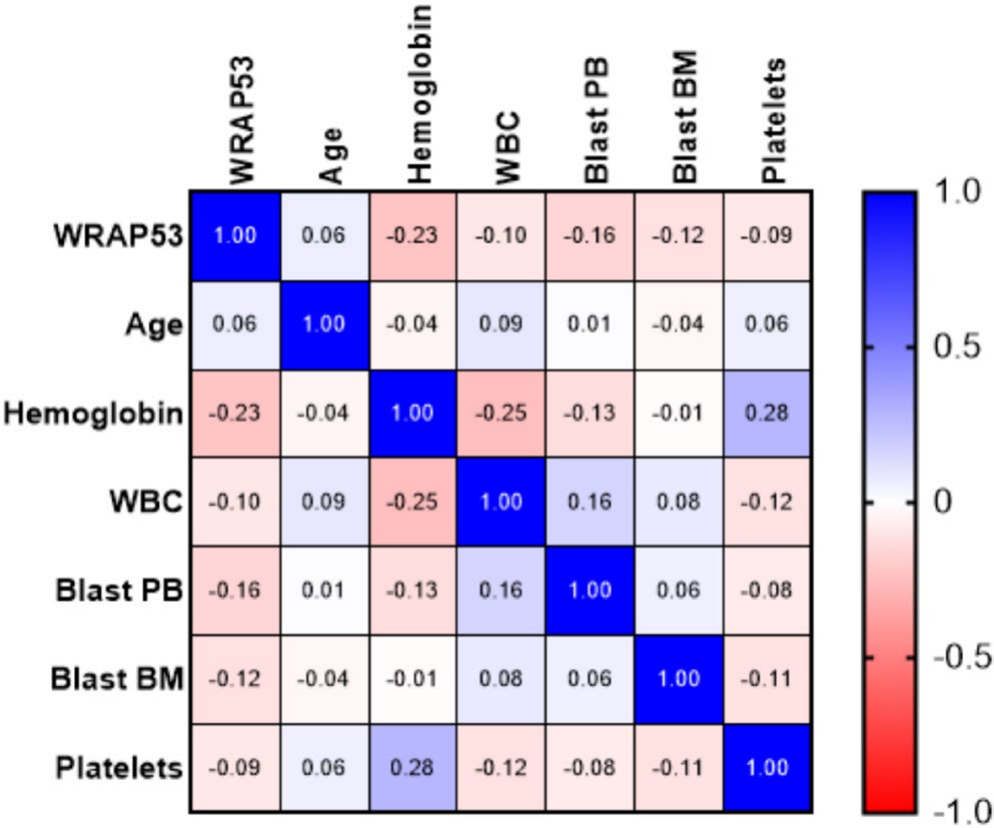
Correlation analysis between *WRAP53* levels and clinical and laboratory parameters in patients with acute leukemia. The color intensity reflects the magnitude of the correlation and the associations between the variables *WRAP53*, age, hemoglobin, WBC, peripheral blood blasts, bone marrow blasts, and platelet count. The values range from −1 (negative correlation) to 1 (positive correlation), with 0 indicating no association between the variables. The _observed variations have correlations ranging from weak to moderate. BM, Bone morrow; PB, Peripheral blood; WBC, White blood cell.

Among the variables analyzed, *WRAP53* showed no correlation with variables, with the most negative values in relation to hemoglobin (−0.23). The other variables analyzed here presented weak correlations with each other, showing limited or independent interactions in the sample analyzed (Figure 6).

**FIGURE 7:**
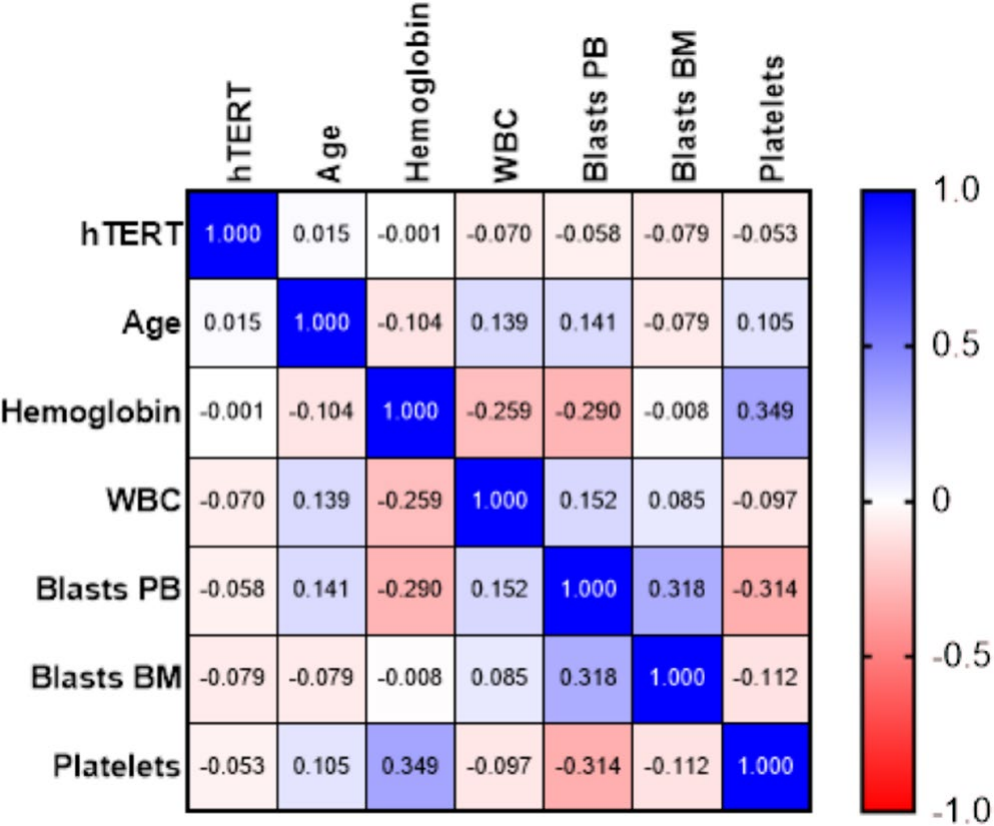
Correlation analysis between *hTERT* levels, clinical and laboratory parameters in patients with acute leukemias. The color intensity reflects the magnitude of the correlation and the associations between the variables *hTERT*, age, hemoglobin, white blood cell, peripheral blood blasts, bone marrow blasts, and platelet count. The values range from −1 (negative correlation) to 1 (positive correlation), with 0 indicating no association between the variables. *hTERT* showed weak correlations with the other variables, while the most notable correlations include the relationship between hemoglobin and platelets (0.35) and between Blast BM and Blast PB (0.32). BM, Bone morrow; PB, Peripheral blood; WBC, White blood cell.

*hTERT* showed very weak correlations with all variables, indicating that this variable is practically independent of the others analyzed. Other variables also showed weak correlations with each other, such as WBC and peripheral blood (PB) blasts (0.15) and Blast Bone marrow (BM) and Blast PB (0.32), indicating a moderate relationship between the latter. On the other hand, it was possible to observe a positive correlation between hemoglobin and platelets (0.35), revealing a moderate association. Hemoglobin also correlated negatively with WBC (−0.26) and Blast PB (−0.29) (Figure 7).

Multiple linear regression analysis revealed a reciprocal association between *WRAP53* and *hTERT* gene expression. When *hTERT* expression was considered as the dependent variable, only *WRAP53* maintained a robust independent effect (*β* = 0.709; *p* < 0.001), explaining approximately 27% of its variability. In contrast, when *WRAP53* was modeled as the outcome, only *hTERT* was significantly positively associated (*β* = 0.282; *p* < 0.001), accounting for approximately 26% of the gene's variability. In both models, clinical variables showed no statistically significant association. The linear regression assumptions were verified and adequately met in both analyses.

## 5 Discussion

AML is the most common acute leukemia in adults and is characterized by high genetic heterogeneity both in diagnosis and in disease progression. Despite advances, treatment still consists of complex chemotherapy regimens combined with bone marrow transplantation for eligible patients. The identification of new biomarkers is essential because, in addition to contributing to a better understanding of the molecular basis of the disease, it may aid in the diagnosis, prognosis, and monitoring of AML [29, 30]. Recent studies indicate that structural alterations or changes in the expression levels of *WRAP53* transcripts may be associated with genomic instability and impaired DNA repair, mechanisms that may contribute to disease progression [8].

In this study, a slightly greater frequency of AML was observed in men, and a greater incidence was observed among individuals aged 55 years or older. Clinical characteristics, such as hemoglobin values, leukocyte counts, the presence of blasts in peripheral blood, and platelet counts, revealed the presence of anemia, thrombocytopenia, and leukocytosis with the presence of circulating blast cells in our patients. These findings are similar to those reported in the literature for patients with AML [31, 32, 33, 34, 35].

The *WRAP53* gene has three transcriptional start sites, from which the transcript variants WRAP53‐α, WRAP53‐β, and WRAP53‐γ are generated, whose expression levels are generally increased in cancer. The α and β isoforms are the most frequently studied in different tumor types. WRAP53α acts as an antisense RNA for the TP53 gene, while WRAP53β encodes a protein that can be localized in both the nucleus and cytoplasm of tumor cells. WRAP53β has been widely investigated due to its potential as a biomarker of both radiotherapy resistance and as a predictor of poor prognosis in several cancers, including colorectal [19], ovarian [22], breast [36], head and neck [37], and lung carcinomas [38, 39, 40]. To date, there are no studies in the literature that have clearly elucidated the biological importance and function of the WRAP53‐γ variant. Furthermore, dyskeratosis congenita, a disease characterized by rapid and progressive shortening of telomeres with a high probability of developing bone marrow failure and cancer, is due to the presence of mutations in the *WRAP53* gene [41]. WRAP53β action is independent of WRAP53α‐mediated p53 regulation [13].

On the other hand, for Bergstrand, Obrien, and Farnebo (2019) [42], the frequently observed high expression of WRAP53β in cancer is related to its involvement in the DNA damage response (DDR) that arises due to precancerous lesions. The authors argue that in response to replication stress and DDR signaling, WRAP53β overexpression occurs, which helps recruit factors that act in DNA repair to maintain genomic integrity. However, cells that accumulate mutations that impair the DDR, such as reduced WRAP53β expression, are able to escape cellular control mechanisms, thus contributing to genomic instability and cancer progression. The main reason for this argument is that most studies reporting WRAP53β overexpression did not observe an association with survival.

In this study, we demonstrated that *WRAP53* expression was lower in the AML patient cohort than in the control cohort (*p* < 0.0001). Studies show that low levels of *WRAP53* associate with downregulation of factors involved in DDR due to downregulation of WRAP53β; moreover, WRAP53α may also be downregulated, which in turn will result in the inactivation of p53 at the mRNA level, thus impairing p53 action after DNA damage [13, 43, 44, 45]. In ovarian cancer, downregulation of WRAP53β or the presence of genetic variations in *WRAP53* is associated with increased mortality and increased risk of ovarian development [22, 46], whereas in metastatic rectal and head and neck cancer, it is related to resistance to radiotherapy [19, 20, 37, 47, 48]. In breast cancer, low nucleolar levels are associated with increased recurrence, death, and resistance to radiotherapy [23, 36]. Moreover, the high presence of nuclear WRAP53β is related to faster and more effective DNA repair due to the rapid recruitment of DDR factors [49]. The action of WRAP53β is independent of and does not influence the regulation of p53 mediated by WRAP53α [13]. The DDR is essential for leukemic cells, but this function is often abnormal in AML, which may contribute to leukemogenesis and treatment resistance, resulting in an unfavorable prognosis [50, 51].

Here, we evaluated the expression of the *WRAP53* gene in combination with clinical factors, such as hematological parameters, risk classification, age and sex, where among these factors, only sex and age presented statistical significance, specifically in the FC < −1 group, where it was observed that females presented a higher mean expression of *WRAP53* (*p* = 0.0322) than males did. A difference in expression was also found in patients over 60 years of age, where this group presented a greater mean than did the ≤ 60 years of age group (*p* = 0.0496). In studies with solid tumors, *WRAP53* expression was not independently analyzed in correlation with all the parameters analyzed here, as it was only paired with age or sex, where no correlations with *WRAP53* expression were observed [36, 38, 40]. The clinicopathological characteristics analyzed in most studies were the degree of tumor differentiation, tumor size, the presence of invasion or lymph node metastasis, and clinical stage (I, II, III, IV, or V), all of which are not applicable for our nonsolid tumor patient cohort and were not analyzed in this work [19, 38, 40, 52].

Regarding the analysis of *hTERT*, in this study, the analysis of gene expression in peripheral blood did not reveal a difference in expression when compared with patients in the control group (*p* = 0.2524). Calvello et al., (2017) [11], when analyzing the expression of *hTERT* in patients with AML, reported a lower total expression of *hTERT*, which was attributed to the presence of the transdominant variant –α + β of *hTERT*, where patients with AML presented a lower percentage of the active isoform of *hTERT* (+α + β) than did the control group. However, other studies indicate that the presence of the active isoform of telomerase in the patients analyzed was greater than 20% [53]. The main *hTERT* isoform described by Kilian et al., 1997 [54] is +α + β, which produces the complete transcript capable of producing the active telomerase enzyme. The deletion of α (−α), which is present in exon 6, acts as a dominant inhibitor of telomerase, and the deletion of ‐β leads to degradation of the mRNA transcript due to the presence of a premature stop codon (*nonsense* codon). Finally, the deletion of both leads to the isoform ‐α‐β, which also results in mRNA degradation due to the presence of nonsense codons. The presence of deletions in α or β generates inactive products to avoid the presence of a truncated and nonfunctional protein [55, 56, 57, 58].

In the present study, we observed three distinct *hTERT* expression profiles among AML patients when compared to healthy donors. While some patients showed reduced or unaltered expression, others displayed moderate expression. This variability may reflect the molecular heterogeneity of AML and suggests differential regulation of telomerase activity across subgroups. Overexpression of *hTERT* may be linked to a greater proliferative potential of leukemic cells, contributing to disease progression and possible treatment resistance [59, 60, 61, 62, 63, 64, 65, 66], while low expression may indicate the activation of alternative telomere maintenance pathways or reflect differentiated stages of leukemogenesis, potentially associated with poor survival [12, 67, 68].

Capraro et al. (2011) [66] reported that *hTERT* expression and telomerase activity follow the order of B‐ALL > T‐ALL > AML, where acute lymphoblastic leukemia (ALL) presents both higher telomerase activity and higher expression of *hTERT* expression than AML does. In addition, telomere length was shorter in AML, T‐ALL, and B‐ALL, respectively. Furthermore, among the subtypes reported for AML, the M0, M3, and M5 subtypes are often mentioned as those with the shortest telomeres and the lowest telomerase expression/activity [10, 66, 67]. These results show how complex the *hTERT* regulatory system can be in acute leukemias [69].

*hTERT* activation is commonly found in hematopoietic cells, germ cells, embryonic stem cells, and cancer cells because of its high replicative power. Altered *hTERT* expression in humans leads to immortality, one of the main hallmarks of cancer [1]. Like many human malignancies, leukemias may exhibit increased telomerase activation, accompanied by elevated *hTERT* expression. Its dysregulation is a common alteration in leukemogenesis, being present from diagnosis to remission, and is found in the 4 main types of leukemia: acute myeloid leukemia (AML), acute lymphoblastic leukemia (ALL), chronic myeloid leukemia (CML), and chronic lymphocytic leukemia (CLL), which are associated with the development and progression of the disease [61, 62, 70, 71, 72, 73, 74, 75]. However, the prevalence of *hTERT* dysregulation is highly variable and dependent on the population of each study [69].

*hTERT* expression was not correlated with WBC count, age, sex, hemoglobin, or risk groups. These findings are in line with other studies that found no association with these parameters [9, 11, 62, 76]. However, its frequent overexpression at diagnosis and relapse reinforces its biological relevance in AML, particularly in association with complex karyotypes and extramedullary disease [9, 53, 61, 62, 64, 65, 77]. In ALL, high telomerase activity and *hTERT* expression are associated with disease progression, treatment resistance, shorter survival, and cytogenetic abnormalities, indicating its potential as a biomarker in adult and pediatric patients [67, 75]. While in CLL, high telomerase expression and activity were able to identify patients with different outcomes and were associated with the presence of short telomeres [73, 74]. In CML, high activity is related to the presence of cytogenetic alterations [78].

The overall survival analysis conducted here revealed no significant difference between the *WRAP53* and *hTERT* Log‐Rank groups: *p* = 0.150; and *p* = 0.316 respectively. This is contrary to what is reported in studies on solid tumors, where high expression levels related to the cytoplasmic presence of WRAP53β or its low nuclear presence are correlated to low overall survival rates [22, 23, 36, 47]. The same is found for *hTERT*, where telomerase expression levels or activity were shown as independent prognostic factors for survival in adults with acute or pediatric leukemia [67, 75].

Pearson correlation analysis between *WRAP53* and *hTERT* genes revealed a positive and moderate correlation coefficient *r* = 0.4374 (*p* < 0.0001). *WRAP53* has previously been correlated with increased telomere length or telomerase activity, but it has not been reported whether telomerase activity was related to prognosis [20, 79, 80]. On the other hand, *hTERT* expression is correlated with telomerase activity and telomere maintenance [66, 81]. This correlation is relevant since high telomerase activity and high *hTERT* expression have been observed in AML patients [61, 65, 82]. Furthermore, chromosomal instability due to progressive telomere shortening has been associated with extremely low telomerase activity in AML and impacts treatment response and survival [66].

Importantly, future studies should explore the molecular mechanisms that regulate the interaction of *WRAP53* with telomerase and other DNA repair components in AML. Furthermore, investigating the subcellular localization of the WRAP53β protein is essential, as it may provide valuable insights into its activity in the cell nucleus or cytoplasm of AML patients. Analyzing these aspects will allow us to better understand the mechanisms by which *WRAP53* may contribute to the development and progression of AML.

## 6 Conclusion

The results presented demonstrate a downregulation of *WRAP53* in the sample of patients studied, indicating that this gene may be involved in other mechanisms related to AML, in addition to its correlation with *hTERT* identified in this study. The reported low expression of *WRAP53* may negatively influence crucial mechanisms that maintain genomic stability, such as the DNA damage response (DDR), which may impact both progression and treatment efficacy in AML due to the accumulation of mutations that confer survival advantages to leukemic cells. Such further investigations may clarify the role of *WRAP53* in the context of AML and its clinical impact, suggesting its potential as a promising molecular biomarker for genomic stability in AML. Furthermore, in this study, *hTERT* expression could not be correlated with the clinical features analyzed.

## Author Contributions

R.B.G., B.M.D.N. and C.A.M.‐N. collected and analyzed data and wrote the manuscript. C.B.M., B.M.D.N., F.M.C.d.P.P., A.K.d.C.M., M.O.d.M.F., M.E.A.d.M., G.S.L., P.H.S.R., H.G.M., D.d.S.O., R.M.R., R.P.G.V., A.S.K. and C.A.M.‐N. provision of data and sub‐sequent analysis and interpretation. Writing – original draft preparation, R.B.G., B.M.D.N. and C.A.M.‐N. writing – review and editing, R.B.G., C.B.M., B.M.D.N., F.M.C.d.P.P. and C.A.M.‐N. All authors have read and agreed to published version of the manuscript.

## Funding

This study was supported by Brazilian funding agencies: Coordination for the Improvement of Higher Education Personnel (CAPES; to B.M.D.N, C.B.M, F.M.C.d.P.P, I.V.B and A.K.C.M), National Council of Technological and Scientific Development (CNPq—PQ scholarships to M.O.d.M.F, A.S.K. and C.A.M.‐N.), Cearense Foundation of Scientific and Technological Support (FUNCAP; to R.B.G).

## Ethics Statement

The study was approved by the ethics committee of the Federal University of Ceará and Dr. César Cals General Hospital with the following approval numbers: 4.339.719 and 5.823.921. The methods were performed in accordance with Helsinki guidelines and regulations.

## Consent

Study patients provided written consent.

## Conflicts of Interest

The authors declare no conflicts of interest.

## Data Availability

The data that support the findings of this study are available from the corresponding author upon reasonable request.
